# Year-round activity levels reveal diurnal foraging constraints in the annual cycle of migratory and non-migratory barnacle geese

**DOI:** 10.1007/s00442-023-05386-x

**Published:** 2023-06-03

**Authors:** Michiel P. Boom, Thomas K. Lameris, Kees H. T. Schreven, Nelleke H. Buitendijk, Sander Moonen, Peter P. de Vries, Elmira Zaynagutdinova, Bart A. Nolet, Henk P. van der Jeugd, Götz Eichhorn

**Affiliations:** 1 Vogeltrekstation—Dutch Centre for Avian Migration and Demography (NIOO-KNAW), Wageningen, The Netherlands; 2 Department of Animal Ecology, Netherlands Institute of Ecology (NIOO-KNAW), Wageningen, The Netherlands; 3 Present Address: Theoretical and Computational Ecology, Institute for Biodiversity and Ecosystem Dynamics, University of Amsterdam, Amsterdam, The Netherlands; 4 NIOZ Royal Netherlands Institute for Sea Research, Den Burg, The Netherlands; 5 Wageningen Environmental Reseach (WEnR), Wageningen, The Netherlands; 6 Institute of Avian Research, Wilhelmshaven, Germany; 7 Institute for Wetlands and Waterbird Research e.V., Verden (Aller), Germany; 8 Department of Vertebrate Zoology, Faculty of Biology, Saint Petersburg State University, St Petersburg, Russia

**Keywords:** Annual cycle, Day length, Foraging, Migration, Residency

## Abstract

Performing migratory journeys comes with energetic costs, which have to be compensated within the annual cycle. An assessment of how and when such compensation occurs is ideally done by comparing full annual cycles of migratory and non-migratory individuals of the same species, which is rarely achieved. We studied free-living migratory and resident barnacle geese belonging to the same flyway (metapopulation), and investigated when differences in foraging activity occur, and when foraging extends beyond available daylight, indicating a diurnal foraging constraint in these usually diurnal animals. We compared foraging activity of migratory (N = 94) and resident (N = 30) geese throughout the annual cycle using GPS-transmitters and 3D-accelerometers, and corroborated this with data on seasonal variation in body condition. Migratory geese were more active than residents during most of the year, amounting to a difference of over 370 h over an entire annual cycle. Activity differences were largest during the periods that comprised preparation for spring and autumn migration. Lengthening days during spring facilitated increased activity, which coincided with an increase in body condition. Both migratory and resident geese were active at night during winter, but migratory geese were also active at night before autumn migration, resulting in a period of night-time activity that was 6 weeks longer than in resident geese. Our results indicate that, at least in geese, seasonal migration requires longer daily activity not only during migration but throughout most of the annual cycle, with migrants being more frequently forced to extend foraging activity into the night.

**Supplementary Information:**

The online version contains supplementary material available at 10.1007/s00442-023-05386-x.

## Introduction

Migration enables animals to exploit seasonally occurring food peaks in different regions throughout the year (Alerstam et al. 2003; Newton 2008; Avgar et al. 2014), or can be a response to changes in resource requirements and pressure from predation and competition during different life stages (Fokkema et al. 2020). Migration itself, however, comes with energetic costs, which have to be balanced within the annual cycle (Buehler and Piersma 2008; Wingfield 2008). By including migration as an additional stage in the annual cycle, migrants face a stronger time constraint compared to residents (Crozier et al. 2008). Moreover, for many species, timing of migration is tightly linked to successful reproduction (Lack 1968; Sedinger and Flint 1991; Prop and de Vries 2007; Post and Forchhammer 2008; Miller-Rushing et al. 2010) through timely arrival with respect to the seasonal peak in food availability and quality (Van der Graaf et al. 2006; Post and Forchhammer 2008; Bischof et al. 2012; Merkle et al. 2016; Ross et al. 2018). The nutritional demands to fuel migration and reproduction follow each other in short succession, while the need to arrive in time at the breeding grounds puts additional time pressure on migratory animals. Thus, in comparison to residents, migrants have additional energetic expenses and are under time pressure to build up their energy stores (Buehler and Piersma 2008).

The ability to fly gives birds unparalleled mobility, enabling them to cover large distances in relatively short periods of time. Active flight, however, is an expensive way of locomotion, resulting in high energetic costs (Alerstam and Bäckman 2018). To fuel their migratory journeys, many migratory birds therefore build up body stores prior to migration (Klaassen 1996; Kvist and Lindström, 2003; Schaub et al. 2008) and during stop-overs (Eichhorn et al. 2006; Rakhimberdiev et al. 2018; Nolet and Drent 1998) by increasing food intake (McWilliams et al. 2004; Eichhorn et al. 2012). This increase in food intake can be achieved by extending the period spent foraging, which has been shown in passerines (Gifford and Odum 1965; Bairlein 2002), shorebirds (Kvist and Lindström, 2003) and geese (Dokter et al. 2018a; Lameris et al. 2021). However, for diurnal birds, foraging time is limited by the available daylight, and when day length is insufficient to meet daily energy requirements they experience a diurnal foraging constraint and may be forced to forage at night (Tinkler et al. 2009; Lameris et al. 2021). Given the energetic expenses of migration, birds that differ in life history strategy (migratory or resident) might therefore differ in the extent to which they experience such diurnal foraging constraints throughout their annual cycle. However, migration to higher latitudes might lift these constraints, because northwards migration is associated with increasing day lengths in spring (Schekkerman et al. 2003; Tjørve et al. 2007; Pokrovsky et al. 2021).

Attempts to look at the compensation for the costs of migration have so far mainly focused on migration distance (Shamoun-Baranes et al. 2017; Weegman et al. 2017), or have been restricted to the migratory period itself (Guillemette et al. 2012; Flack et al. 2016). However, a migratory life history strategy influences the entire annual cycle due to potential carry-over effects (Harrison et al. 2011), and its impact must therefore be evaluated within the complete annual cycle (Marra et al. 2015). Ideally, comparisons between migrants and residents are made within the same species, because species of different phylogenetic background are likely to differ in many other aspects besides life history strategy alone (Garland and Adolph 1994).

For this study, we combined year-round tracking and accelerometery data of barnacle geese from two populations, one resident population breeding along the North Sea coast and one long-distance migratory population breeding in Arctic Russia and wintering along the North Sea coast, and compared their (foraging) activity throughout the whole annual cycle. In search for diurnal foraging constraints experienced by the geese, we identified periods when activity exceeded the available daylight period, which suggests nocturnal foraging. Finally, we present data on seasonal variation in body condition of geese from the same migratory and resident population in support of our conclusions based on activity records.

## Methods

### Study species and populations

Barnacle geese are originally Arctic-breeding migratory birds, and the majority of the population breeds in Arctic Russia along the Barents Sea coast, while wintering along the North Sea coast in South-western Denmark, Northern Germany and the Netherlands (Fox and Leafloor 2018). Following a rapid population increase (Rozenfeld et al. 2021), barnacle geese have expanded their breeding area towards the southwest within the flyway, and have established new populations in the Baltic on Gotland in 1971 and along the North Sea coast in the Netherlands in 1982 (Van der Jeugd et al. 2009). Barnacle geese breeding in these new populations also changed their life history strategy, with Baltic breeders having a greatly reduced migration distance relative to Arctic-breeding geese, and geese breeding along the North Sea coast having become residents. All populations still share the same wintering grounds. This breeding range expansion and coinciding change of life history strategy offers the opportunity to compare activity of migratory and resident birds within the same species.

### GPS-ACC data

We gathered accelerometer (ACC) and GPS data from migratory (n = 94) and resident (n = 30) barnacle geese. We used data from four types of GPS-ACC transmitters, collected between 2014 and 2020; type A: UvABiTS (Bouten et al. 2013), type B: Ornitela (OrniTrack-25), type C: Milsar Technologies S.R.L (GSMRadioTag custom), type D: Madebytheo (Solar GPRS/GPS). Both tracking period and transmitter type overlapped between the migratory and resident populations (Table S1). GPS-ACC transmitters (18.5-25 g) were all attached to adult female geese using a 16-g Teflon harness (Lameris et al. 2017), with the combined weight of transmitter and harness being < 3% of the average female body weight (1615 g; Boom et al. 2022). The use of this harness did not appear to affect migratory behaviour (Lameris et al. 2018). Geese of the migratory population were caught in the breeding colony at Kolokolkova Bay, Russia (68°34′N, 52°18′E), on the nest in 2014 (n = 24) and during post-breeding wing-moult in 2018 (n = 3). Additionally, migratory geese were caught on the wintering grounds using canon-nets in Lower Saxony, Germany (n = 29) and in the province of Fryslân, the Netherlands (n = 36) during the winters of 2016–2020. These birds were assigned to the migratory population when the collected GPS-tracking data confirmed migration.

Birds of the resident population were caught on the nest (n = 23) in the breeding colony at the Westplaat Buitengronden (51°47′N, 4°08′E) in 2015 and 2016 as well as during post-breeding wing-moult in 2018 (n = 7). All geese were measured at capture (body mass, head length, tarsus length, wing length, p9 length, see van der Jeugd et al. (2003) for details) and were equipped with coloured PVC leg rings with inscription for individual recognition.

All GPS-ACC transmitters recorded accelerometer measurements in bursts, with transmitter types differing in burst length (from 0.5 to 5 s) and within-burst frequency (between 20 Hz and 50 Hz). Transmitters took accelerometer bursts on regular time intervals varying between 5–30 min and recorded GPS-positions at intervals varying between 5 and 240 min, both depending on transmitter type and battery level. To keep transmitters with different sampling regimes comparable, we resampled all ACC data to intervals of 30 min. Incomplete days (with < 48 ACC measurements) were excluded from the analysis. Every ACC measurement was matched with the GPS position that was taken closest in time (mean deviation and SD: 17 ± 8 min from ACC measurement).

For every GPS position we calculated day length based on the sunrise and sunset times using the R-package “suncalc” (Thieurmel and Elmarhraoui 2019), in which we defined day length as the period between dawn and dusk (including the period of civil twilight).

### Body mass data

Data on body mass of a larger sample of untracked adult females in both populations was collected in the Netherlands and Russia during various catches over the period 1979–2020 (n = 2744 birds), as well as from shot birds (n = 320) to compare body condition dynamics throughout the year. Data on body mass was collected over the full annual cycle in both populations (see Table S2). Geese captured or shot in the SW part of the Netherlands were considered to belong to the resident population (see supplementary material), as well as all geese caught in the Netherlands in July. Birds captured or shot in the North of the Netherlands, along the migratory route and in the Arctic were considered to belong to the migratory population. Body condition was defined as body mass corrected for size (head length) using the scaled body mass index, calculated following Peig and Green (2009) with mean head length = 81.25 mm and b_SMA_ = 3.37 as coefficients.

### Data analyses

#### Activity classification

We used ACC data to classify activity budgets for individual geese. To deal with the differences in accelerometer types as well as burst length and burst frequency, we used the vectorial sum of dynamic body acceleration as measure of activity (Qasem et al. 2012; Dokter et al. 2018b). The vectorial sum of dynamic body acceleration was calculated for each burst by taking the square root of the summed variances on all three accelerometer axes (x, y, z; surge, sway, heave) which measure acceleration in g0 (standard gravity). For every transmitter type separately, we then created probability density histograms. Based on these histograms we determined the peaks for inactive, active and flying behaviour (Dokter et al. 2018b). We used the “mix” function in the R-package “mixdist” (Macdonald and Du 2018) to unravel the gamma distributions that make up the probability histogram. We instructed the function to assume two underlying gamma-distributions (for active and inactive behaviour). By calculating the intersections of the distributions for inactive and active behaviour, we determined transmitter-specific thresholds distinguishing active from inactive behaviour (Fig. S1). Flying behaviour was determined as VeDBA > 550. Further analyses are focused on active behaviour on the ground only (hereafter simply referred to as “activity”), which serves as a proxy for foraging behaviour since foraging takes up over 80% of the active behaviour observed in wild geese (Drent et al. 1978; Owen et al. 1992).

### Correction for breeding timing

**Fig. 1:**
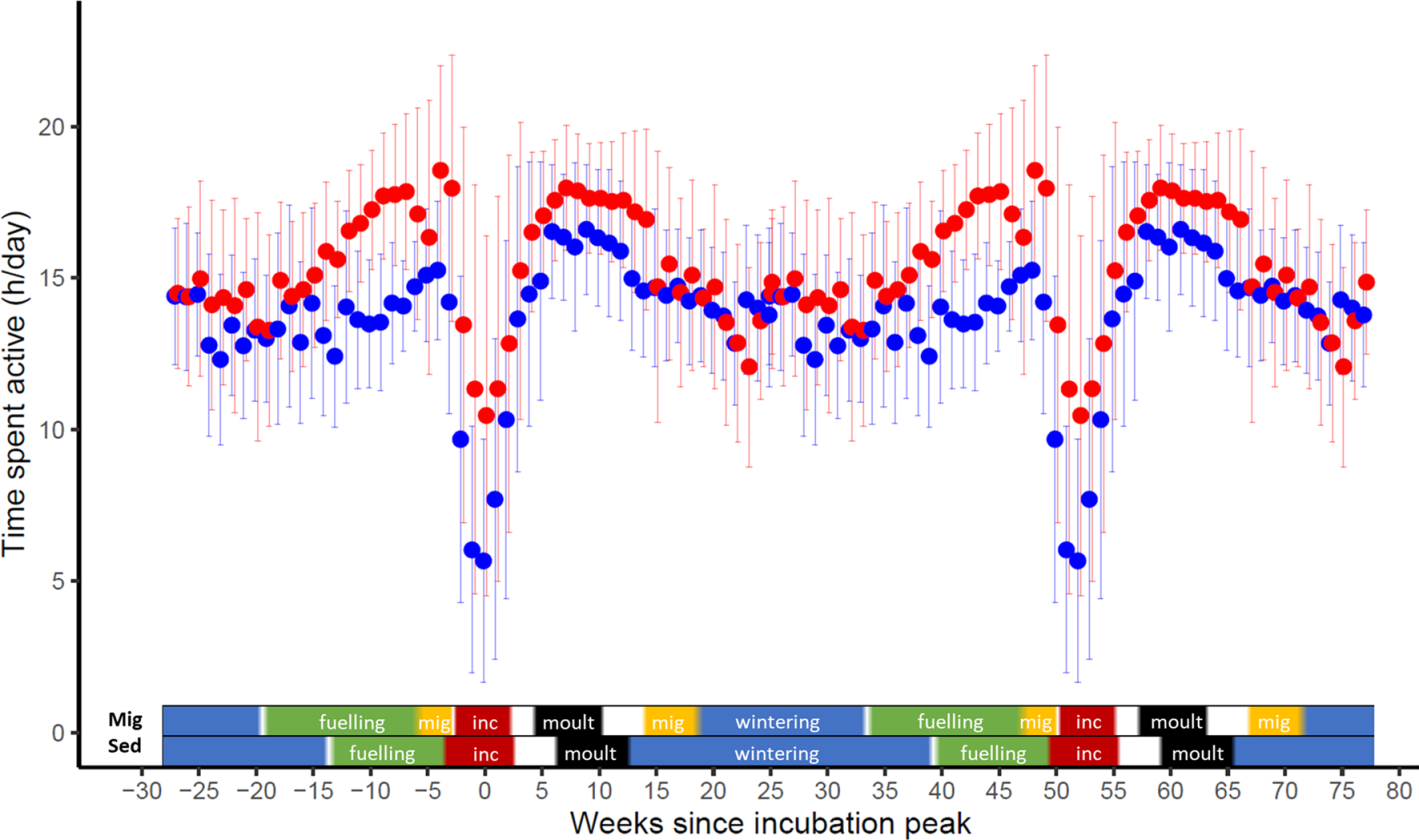
Double plot (i.e. the annual cycle is repeated for illustrative purpose) of the variation in time spent active throughout the year (weekly means ± SD) for barnacle geese (*Branta leucopsis*) of the migratory (red) and resident population (blue). Coloured bars above the x-axis show the stage in the annual cycle of both populations (Mig: migratory, Sed: resident), based on data published in van der Jeugd et al. (2009) for incubation (inc; red) and moult (period between mean onset and end of breeding and moult ± 1 SD). Migration periods (mig; yellow) are estimated based on the GPS data. Fuelling and wintering are estimated as the periods between stages with known timing. Blanks indicate uncertainty on life stage. The migratory population is generally more active than the resident year-round

Because of their different life history strategies, the two barnacle goose populations we studied differed in timing of breeding and moult (Van der Jeugd et al. 2009), with the resident population breeding c. 7 weeks earlier and moulting c. 2–4 weeks earlier. To account for the different breeding phase within the annual cycle in our population comparison, we used the distinct trough in activity levels observed during incubation in each population (Fig. 1, Fig. S2). We fitted quadratic curves to the weekly activity data during the breeding period, to determine the moment of minimum activity in each population corresponding to the moment when most geese are incubating, or peak incubation (Fig. S2). We then centred the annual cycle of each population relative to this moment of peak incubation. Within this approach, we did not distinguish between breeding and non-breeding individuals since we could not determine breeding for all birds included in this study because not all tracked birds provided (sufficient) data during the breeding period.

### Determining periods of activity differences

To quantify to what extent and when in the annual cycle the populations differed in activity, we compared cumulative activity throughout the annual cycle. To do so, we first averaged daily activity per week to smooth out fluctuations due to days on which only a few transmitters provided a complete day of data: For each day in a week, we averaged the time spent active over the individuals of the resident and migratory population respectively that provided a complete day of data. Subsequently we calculated the daily average over each week. By first averaging over day, we make sure every day is treated equally in the analysis, regardless of the number of individuals that provided data that day. Based on these weekly averages we calculated the cumulative difference in mean daily activity per week between the resident and migratory population over the year. Calculating the cumulative activity difference makes it possible to identify the periods in which similar activity differences exists, while also demonstrating what these differences accumulate to over the year. We excluded the incubation period (determined based on published periods in Van der Jeugd et al. (2009)) from this analysis, because potential population differences in the proportion of incubating birds may influence the mean activity of the population. To study whether differences in activity between populations change throughout the year, we fitted segmented linear regressions to identify breakpoints using the package “segmented” in R 4.0.1 (Muggeo 2008; R Development Core Team 2020). The cumulative difference in activity was used as dependent variable and week as independent variable. We used the Bayesian Information Criterion (BIC) to compare models with different numbers of breakpoints (D’angelo and Priulla 2020). Segments with positive slopes then indicate periods of higher activity of the migratory population, whereas negative slopes refer to periods with a higher activity in the resident population.

In addition, we compared the average body condition (scaled body mass index, see “Body mass data”) of the populations to determine if periods with differences in activity coincided with differences in body condition. Body condition was grouped per month and population to ensure sufficient body mass data coverage of both populations throughout the year. We could not correct for annual differences in body condition, because birds were caught at different times of the month in different years (e.g. beginning or end of the month). By grouping body condition measures of all years per month, such differences were smoothed out. Individuals captured in multiple years and/or months were only included once. We used Mann Whitney U tests to test for differences in body condition between the populations in each month, using a Bonferroni correction for multiple testing.

### Daylight and activity differences

Like daily activity, available daylight was averaged per week. To test whether any differences in activity between populations are explained by differences in day length, we created linear regressions for each segment of the segmented regression analysis described above, in which the difference in activity was used as dependent variable and the difference in day length as independent variable. The population that experienced longer days was expected to be more active, hence the relation between the day length difference and activity difference was expected to be positive. Therefore, we opted for one-sided t-tests to test for an effect of day length difference on the difference in activity.

### Determining diurnal foraging constraints

Because barnacle geese are mainly diurnal birds (Eichhorn et al. 2021), we assumed that night-time foraging occurs when energy requirements cannot be met by daytime foraging alone (Lameris et al. 2021). To determine when such diurnal foraging constraints occur, and whether this differs between populations, we determined when daily activity of geese of either population exceeded day length, thus assessing the active time exceeding day length (AED).

## Results

### Periods of activity differences

Annual cycle patterns of active behaviour (excluding flight) broadly resembled each other, with both populations showing increasing activity in spring culminating in a peak just before incubation. However, the migratory population consistently showed elevated levels of activity compared to the resident population especially in the weeks before and after the incubation period (Fig. 1). At the moment of spring migration (the first migration period in Fig. 1, indicated with yellow in the bar showing the annual cycle stages), activity of the migratory population showed a small dip, caused by long periods of flight, but still remained higher than the activity of the resident population.

**Fig. 2:**
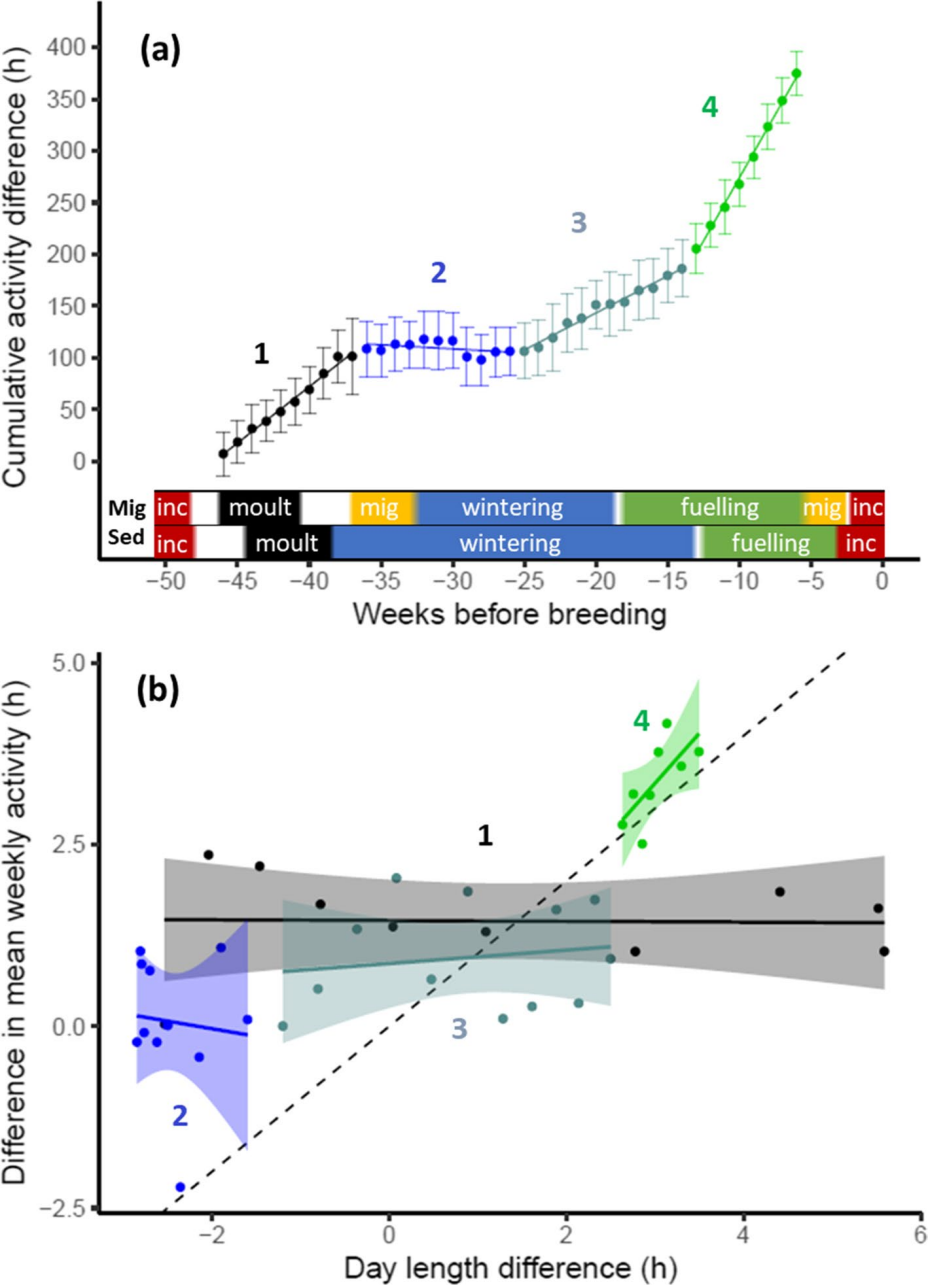
Differences in activity between the migratory and resident population throughout the annual cycle. **a** depicts the cumulative difference in activity per week (mean ± SD of the difference), including four regression lines based on segmented linear regression, indicated by different colours and numbers (see main text for slopes, Table 1 for model selection results). Coloured bars above the x-axis show the stage in the annual cycle of both populations (Mig = migratory, Sed = resident; see legend Fig. 1). **b** shows the relation between the difference in mean weekly activity (mean daily activity per week) and the difference in day length (mean day length per week) between the migratory and resident population for each segment. Regression lines and confidence intervals (shaded bands) are shown based on linear regressions (see main text for slopes). Colours and numbers correspond to the colours of the segments in **a**. The dashed line shows the y=x relationship. The positive slope in period 4 shows that the population difference in activity was partly explained by the longer days experienced by the migratory population in this period

The segmented regression used to investigate activity differences between the migratory and resident barnacle geese indicated three breakpoints in the cumulative difference in weekly activity: at week − 36 (− 36.19 ± 0.36), week − 25 (− 25.37 ± 0.450) and week − 13 (− 13.42 ± 0.25; estimate ± SE) relative to peak incubation (Table 1). These breakpoints split the annual cycle into four different periods of, respectively, (1) week − 46 till − 37, (2) week − 36 till − 26, (3) week − 25 till − 14, and (4) week − 13 till − 6 relative to peak incubation (Fig. 2a). The first period, the post-incubation period including wing moult and preparation for autumn migration, indicated higher mean daily activity for the migratory population (slope = 10.87 mean h/week; t_9_ = 20.97; *P* < 0.001). For the second period, including autumn migration and the beginning of wintering, we found no difference in activity between the populations (slope = − 0.79 mean h/week; t_10_ = − 1.77; *P* = 0.11). During the third period, which includes the rest of the wintering period and passes into the start of the spring fuelling period, activity was again higher in the migratory compared to the resident population (slope = 7.14 mean h/week; t_11_ = 18.14; *P* < 0.001). Similarly, during the fourth period, which covers spring fuelling, a higher mean daily activity was found for the migratory population (slope = 24.39 mean h/week; t_7_ = 33.57; *P* < 0.001). Over one complete annual cycle (excluding the breeding period), the difference in mean daily activity per week accumulated to more than 370 h that the migratory population was more active than the resident population (Fig. 2a).

**Table 1:** Model selection results of the segmented regression with the cumulative difference in mean weekly activity between the migratory and resident population as dependent variable

Model	df	AIC	BIC
~1	2	324.87	328.30
~week	3	260.31	260.31
~week + sl.1 + bp.1	5	188.17	196.73
~week + sl.1 + bp.1 + sl.2 + bp.2	7	162.54	174.54
**~week + sl.1 + bp.1 + sl.2 + bp.2 + sl.3 + bp.3**	**9**	**92.94**	**108.36**
~week + sl.1 + bp.1 + sl.2 + bp.2 + sl.3 + bp.3 + sl.4 + bp.4	11	92.60	111.45

Six candidate models are compared: intercept only (~1), weeks before peak incubation (~week) and 4 models with increasing number of breakpoints (bp.#) and additional slopes (sl.#). The best model (including 3 breakpoints) based on BIC is given in *bold*

We found a higher body condition in the migratory population than in the resident population in the 2 months prior to the moment of peak incubation (month − 2: W = 1619, *P* < 0.001; month − 1: W = 1883.5, *P* < 0.001; Fig. S3). In contrast, during incubation, body condition of the migratory population was lower than the resident population (month 0: W = 5714, *P* < 0.01, Fig. S3). In the 2 months after the moment of peak incubation, body condition was again higher in the migratory population (month 1: W = 5920.5, *P* < 0.001; month 2: W = 55498, *P* < 0.001; Fig. S3).

### Daylight and activity differences

The population difference in activity was only explained by the longer days experienced by the migratory population during the fourth period (i.e., spring fuelling) (t_6_ = 2.434, *P* < 0.05). With an increasing difference in daylight of 1 h, the difference in activity between the populations increased by 1.38 h (Table 2, Fig. 2b). Daylight did not explain activity differences between the populations in the other three periods (period 1: t_8_ = − 0.068*, P* = 0.48; period 2: t_9_ = − 0.28, *P* = 0.40; period 3: t_10_ = 0.51, *P* = 0.31; Table 2; Fig 2b).

**Table 2:** Relation between the difference in mean weekly activity and difference in day length between the migratory and resident population for the four different segments of the segmented regression analysis

Segment	Model	Estimate	SE	t-value	*P*-value
Segment 1	**Intercept**	**1.46**	**0.24**	**5.96**	**<0.001**
	Day length difference	− 0.00059	0.076	− 0.068	0.48
Segment 2	Intercept	− 0.45	1.85	− 0.24	0.81
	Day length difference	− 0.21	0.74	− 0.28	0.40
Segment 3	**Intercept**	**0.87**	**0.27**	**3.15**	**0.01**
	Day length difference	0.092	0.18	0.51	0.31
Segment 4	Intercept	− 0.78	1.71	− 0.47	0.66
	**Day length difference**	**1.38**	**0.57**	**2.43**	**0.026**

P-values for slopes are one-sided, P-values for intercepts are two-sided. Significant effects are indicated in bold

### Daylight and diurnal foraging constraints

**Fig. 3:**
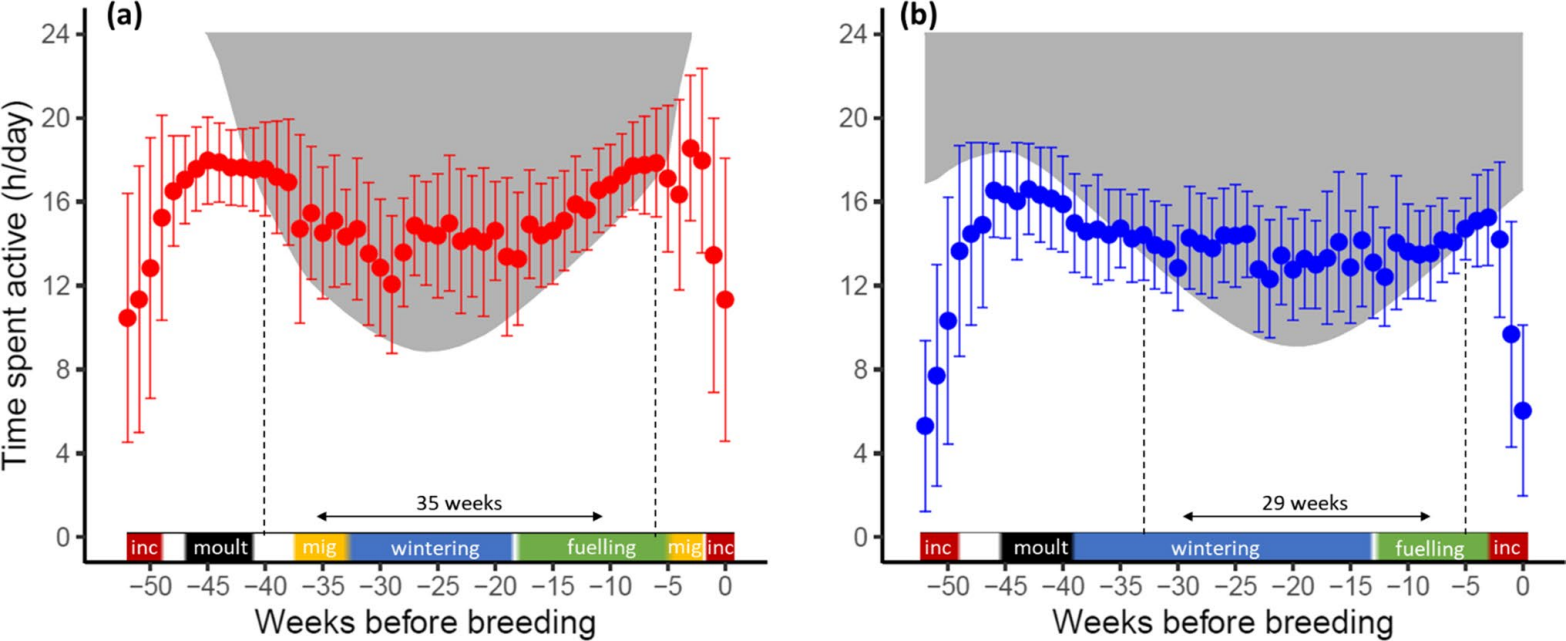
Daily activity per week (mean ± SD) for the migratory (panel **a**, red) and resident population (panel **b**, blue), in relation to the available day length (indicated by the shaded area). The shaded area represents the night (period between dusk and dawn), points within the shaded area indicate activity exceeding available daylight (AED). The dashed vertical lines indicate the period during which AED occurs (i.e., is positive). Coloured bars above the x-axis show the stage in the annual cycle of both populations (see legend Fig. 1). The migratory population faces a longer diurnal foraging constraint than the resident one, particularly in autumn

Individuals from both populations were found to be active for longer than the day length (i.e., AED > 0) during the non-breeding part of the year (Fig. 3a, b). AED in the migratory population started to occur shortly after wing moult and continued until spring migration. In the resident population, AED lasted from approximately 1 month after moult until about 2 weeks prior to the onset of incubation. The period with AED was longer in the migratory population by an additional 6 weeks, and included the post-moult and autumn migration (− 40 till − 33 weeks relative to peak incubation).

## Discussion

We examined whether migratory barnacle geese show more foraging activity, here measured as overall activity excluding flight, than resident barnacle geese, and when differences in activity occur in the annual cycle. We found that the migratory population showed higher activity throughout most of the year, particularly in the periods preceding spring and autumn migration. Although our measure of activity does not immediately translate to foraging, observational studies on barnacle geese confirmed that, of the period spent active, most of this time (80–90%) is allocated to foraging (Ebbinge et al. 1975; Black et al. 1991; Owen et al. 1992). Below we discuss the role of foraging activity in balancing the energetic costs of migration, and if this is mediated by varying day length.

### Differences preceding spring migration

The population difference in activity was largest in the period preceding spring migration, when the migratory population was, on average, 3.4 h/day longer active than the resident population. While we could not distinguish between breeding and non-breeding birds, activity differences might exist, especially in spring. Breeding probability increased with increasing foraging time during spring migration in white-fronted geese (*Anser albifrons*) (Cunningham et al. 2023). The observed activity difference during peak incubation, being higher in the migratory than resident population (Fig. 1), suggests a lower breeding propensity in the migratory population, which would mean that the activity difference in spring would be even larger when only considering breeding birds.

The increased activity preceding migration probably underlines the need to acquire sufficient body stores prior to migration. Increased (foraging) activity in preparation for migration has been reported in passerines, shorebirds and waterfowl (Gifford and Odum 1965; Bairlein 2002; Kvist and Lindström, 2003; Dokter et al. 2018a). For example, white-crowned sparrows increased foraging time when approaching migration by becoming active throughout the whole daylight period (instead of activity peaks in the morning and evening) (Ramenofsky et al. 2003), while shorebirds and waterfowl are also able to forage nocturnally to increase fuelling rates (Zwarts et al. 1990; McNeil et al. 1992; Lameris et al. 2021). In line with the observed increase in foraging activity, body condition of barnacle geese increased during spring, with a higher peak body condition in spring in the migratory population (prior to spring migration) compared to the resident population (prior to incubation) (Fig. S3). Geese are partly capital breeders that accumulate body stores to fuel egg formation and incubation (Drent et al. 2007; Hahn et al. 2011). Part of these stores are deposited prior to migration and replenished during stopovers on the way to the breeding grounds. The transport of body stores is considered energetically expensive, increasing the costs of migration (Pennycuick 1989; Hedenström and Alerstam 1997). Moreover, most barnacle geese currently bypass intermediate spring staging sites in the Baltic and transport an overload of body stores to fly directly from the Wadden Sea to the distant Arctic (see Eichhorn et al. (2009) for an estimate of costs incurred by this strategy). The transportation costs of extra body stores for fuelling long-distance flights and reproduction may contribute to the larger differences in both activity and body condition between individuals of the migratory and resident population prior to spring migration, as compared to autumn, when the difference between the populations is presumed to be mainly caused by the preparation for migratory flight (also see Kölzsch et al. 2016).

### Day length and diurnal foraging constraints

The lengthening of days in spring is known to facilitate increased foraging activity in animals (Kvist and Lindström, 2000; Hill et al. 2003; Pokrovsky et al. 2021), and due to the northward movement, the migratory population experiences longer days during spring migration as compared to resident geese. As they move North, barnacle geese quickly adjust their circadian rhythm and thereby take full advantage of longer daylight by prolonging their active phase (Eichhorn et al. 2021). The activity levels of the migratory population are similar to white-fronted geese migrating along the same flyway, in which daily activity ranged from 14.6 to 21 h (compared to 14.4–19.3 h in our study) (Pokrovsky et al. 2021).

However, we found that differences in activity levels between migratory and resident barnacle geese were not fully explained by differences in day length. This is especially clear prior to autumn migration, when day length decreases and differences in day length did not facilitate the higher activity of the migratory population. Despite the stronger decrease in day length in the Arctic, the activity difference between the migratory and resident population remained constant and the activity of the migratory population even started to exceed day length. In line with this observation, Eichhorn et al. (2021) reported that migratory barnacle geese in the Russian Arctic were arrhythmic not only during the Polar day (24 h light), but remained so for nearly 1 month after the Polar day had ended. In autumn, food quality is declining as a result of plant aging (Lindholm et al. 1994; Van der Graaf et al. 2006), which could further drive migratory geese to be foraging for longer periods of time to meet energy demands. In contrast, resident geese can profit from high quality food year round, as a result of agricultural intensification (Abraham et al. 2005; Eichhorn et al. 2012), which might allow for lower levels of foraging activity (Dokter et al. 2018b; Pot et al. 2019). Although migratory geese might experience relatively lower food quality in autumn, they have access to high quality food on agricultural pastures in winter (Pot et al. 2019) and food quality experienced by resident and migratory geese was found to be comparable during spring and breeding (van der Jeugd et al. 2009). It seems therefore unlikely that the observed activity differences outside autumn are caused by differences in food quality.

Activity of both populations exceeded the available day length during the non-breeding period. Because activity of migratory geese already started to exceed the day length when preparing for autumn migration at Arctic staging sites, the total AED period for the migratory population was 6 weeks longer than for residents. Animals are expected to experience a diurnal foraging constraint particularly in winter, when days are shortest, food quality and abundance drops, and lower temperatures cause higher thermoregulation costs. In line with findings of Owen et al. (1992) and Prop (2004), activity of barnacle geese exceeded the day length most markedly mid-winter, when days are shortest. During this period, barnacle geese use moonlit nights for nocturnal foraging (Ydenberg et al. 1984), which also happens further into spring, but to a decreasing extent (Lameris et al. 2021). Our measure of activity does not immediately translate to foraging, and part of the AED might therefore consist of other behaviours which might explain the large amount of AED. It is possible that at night, geese allocate more time to other active behaviours like preening rather than foraging, which we could not disentangle with our approach. However, this is the case for both populations and the differences in AED thus remain. In spring, activity of both populations continued to exceed the available day length. During this period both populations are preparing either for breeding (resident population) or migration and breeding (migratory population). By then, (part of) the migratory geese are already experiencing longer days at northern stopovers than the resident geese, therefore absolute activity is higher in the migratory population, probably also due to the need to accumulate body stores for spring migration on top of the stores for breeding. One may expect that different levels of food processing rate and physical activity (including migratory flights) as observed in resident and migratory geese go hand in hand with physiological adjustments. Eichhorn et al. (2019) indeed found a higher basal metabolic rate in the migratory geese (measured during summer post-breeding), which likely reflects a larger ‘metabolic machinery’.

### Two viable strategies

Although we show that a migratory life history strategy comes with diurnal foraging constraints for adults, this is likely to be offset by benefits for the offspring (Lack 1968). Correspondingly, goslings in the migratory population grow faster (Boom et al. 2022) and experience higher pre-fledging survival than goslings in the resident population (Fokkema et al. 2020). This illustrates the benefits of migrating to the Arctic to breed, which is in line with findings in other Arctic breeding birds such as waders (Schekkerman et al. 2003). On the other hand, clutches laid by resident breeding barnacle geese are, on average, one egg larger than those produced in the Arctic-migratory population (Eichhorn et al. 2010) and post-fledging survival is markedly higher in the resident population (Fokkema et al. 2020; van der Jeugd et al. 2009). Overall, the population growth rate of the migratory population was found to be positive (1.034), albeit lower than for the resident population (1.139), showing that both strategies are currently viable (Fokkema et al. 2020). Although the resident strategy currently appears to be the better option, it is important to note that the resident population is likely still in an earlier stage of population development, where density dependent effects have not yet set in. Furthermore, when considering the size of available breeding habitat, it is highly unlikely that the breeding area of the resident geese could sustain similar numbers as the breeding areas of the migratory population in the Arctic.

## Conclusions

In a changing environment the balance between the costs and benefits of migration might change, causing migration to evolve or be suppressed in populations (Alerstam et al. 2003). Obtaining an overview of the mechanisms that contribute to this balance is required to gain insight into the adaptations that co-occur with migration. By comparing seasonally migratory and resident populations of barnacle geese, we show that a migratory life history strategy involves a higher amount of daily (foraging) activity not only during periods of active migration but year-round, most notably in the periods preceding migration. While spring migration has been a prominent focus in migration research (Newton 2008), the potential challenges for migratory birds preparing for autumn migration are less clear. In spring, lengthening days and improving food conditions (both coinciding with latitudinal movement) aid migratory birds to meet their energy requirements (Pokrovsky et al. 2021; La Sorte and Graham 2021; van der Graaf et al. 2006). In contrast, the need to prepare for migration under the shortening days and declining food quality in autumn forces migratory geese to be active beyond the available day length. While the post-breeding period is known to be important for juvenile survival in migratory birds (Owen and Black 1989; Rotics et al. 2016; Jones and Ward 2020), our results show that this period might also be crucial for adults, because fuelling for autumn migration occurs under deteriorating conditions.

## Supplementary Information

Below is the link to the electronic supplementary material.

Supplementary file1 (DOCX 522 KB)

## Data Availability

Data will be made available through the dryad digital data repository.
