# Supplementary material for: Year-round activity levels reveal diurnal foraging constraints in the annual cycle of migratory and non-migratory barnacle geese
Source: Oecologia. 2023 Jun 3;202(2):287–98. doi: 10.1007/s00442-023-05386-x (PMC10307695; doi:10.1007/s00442-023-05386-x)
Supplement: Supplementary file 1 — Supplementary file1 (DOCX 522 KB) [file 442_2023_5386_MOESM1_ESM.docx]

**Electronic Supplementary Material**

**body mass data collection**

Depending on the time of year, geese were caught 1) with spring traps on the nest in the breeding period (Netherlands and Russia); 2) by driving geese in a catching pen during the flightless period of post-breeding wing-moult (Netherlands and Russia) or 3) by using canon- or whoosh nets (winter and spring, in the Netherlands, Germany, Denmark and Sweden). During these catches, geese were measured (head length, tarsus length, wing length), and body mass was recorded to the nearest 10 g. In addition to body mass collected from captures (n=3179), a further 316 birds that were shot in spring and autumn (in 2003-2004 and 2014-2018) during regular hunting activities were measured and weighed. Geese captured or shot in the SW part of the Netherlands (SW of the line between Rotterdam and Tilburg) were considered to belong to the resident population, as well as all geese caught in the Netherlands in July. Geese shot North of the city of Zwolle were considered to belong to the migratory population. During the winter months (December, January, February, March) when wintering distributions overlap, over 45% of the geese present in the SW part of the Netherlands are considered residents (Fauna Beheer Eenheid Zuid-Holland, 2021). During catches in winter in the SW part of the Netherlands we intentionally targeted groups with ringed resident birds to further increase the chance of capturing resident birds. Likewise, in the SW of the Netherlands we specifically weighed birds shot in the vicinity of the breeding grounds during regular hunting activities, which are most likely to belong to the local resident breeding population. Nonetheless, body mass patterns in the winter period should be interpreted with caution.

**Tables**

Table S 1: Overview of the number of barnacle geese equipped with transmitters of the 4 different types (A, B, C and D, see main text) over the study period from 2014 to 2020.

|  | **Migratory population** | | | | |  | **Resident population** | | |
| --- | --- | --- | --- | --- | --- | --- | --- | --- | --- |
| **Year** | **Type A** | **Type B** | **Type C** | **Type D** | **Total** |  | **Type A** | **Type C** | **Total** |
| 2014 | 24 |  |  |  | 24 |  |  |  |  |
| 2015 | 24 |  |  |  | 24 |  | 6 |  | 6 |
| 2016 |  |  |  | 31 | 31 |  | 23 |  | 23 |
| 2017 |  |  |  | 32 | 32 |  | 15 |  | 15 |
| 2018 |  | 3 | 3 | 22 | 28 |  | 2 | 7 | 9 |
| 2019 |  | 29 | 2 | 14 | 45 |  | 1 | 3 | 4 |
| 2020 |  | 34 |  | 11 | 45 |  |  | 2 | 2 |

*Table S 2: Number of adult female geese per population and month (relative to the incubation period) that were used to compare body condition dynamics throughout the year.*

|  | **Population** | |
| --- | --- | --- |
| **Month  (relative to incubation period)** | **Resident** | **Migratory** |
| -5 | 8 | 361 |
| -4 | 43 | 177 |
| -3 | 28 | 89 |
| -2 | 80 | 88 |
| -1 | 58 | 115 |
| 0 | 67 | 128 |
| 1 | 28 | 742 |
| 2 | 474 | 336 |
| 3 | 0 | 0 |
| 4 | 22 | 1 |
| 5 | 8 | 21 |
| 6 | 0 | 190 |
| **Total** | **816** | **2248** |

**Figures**


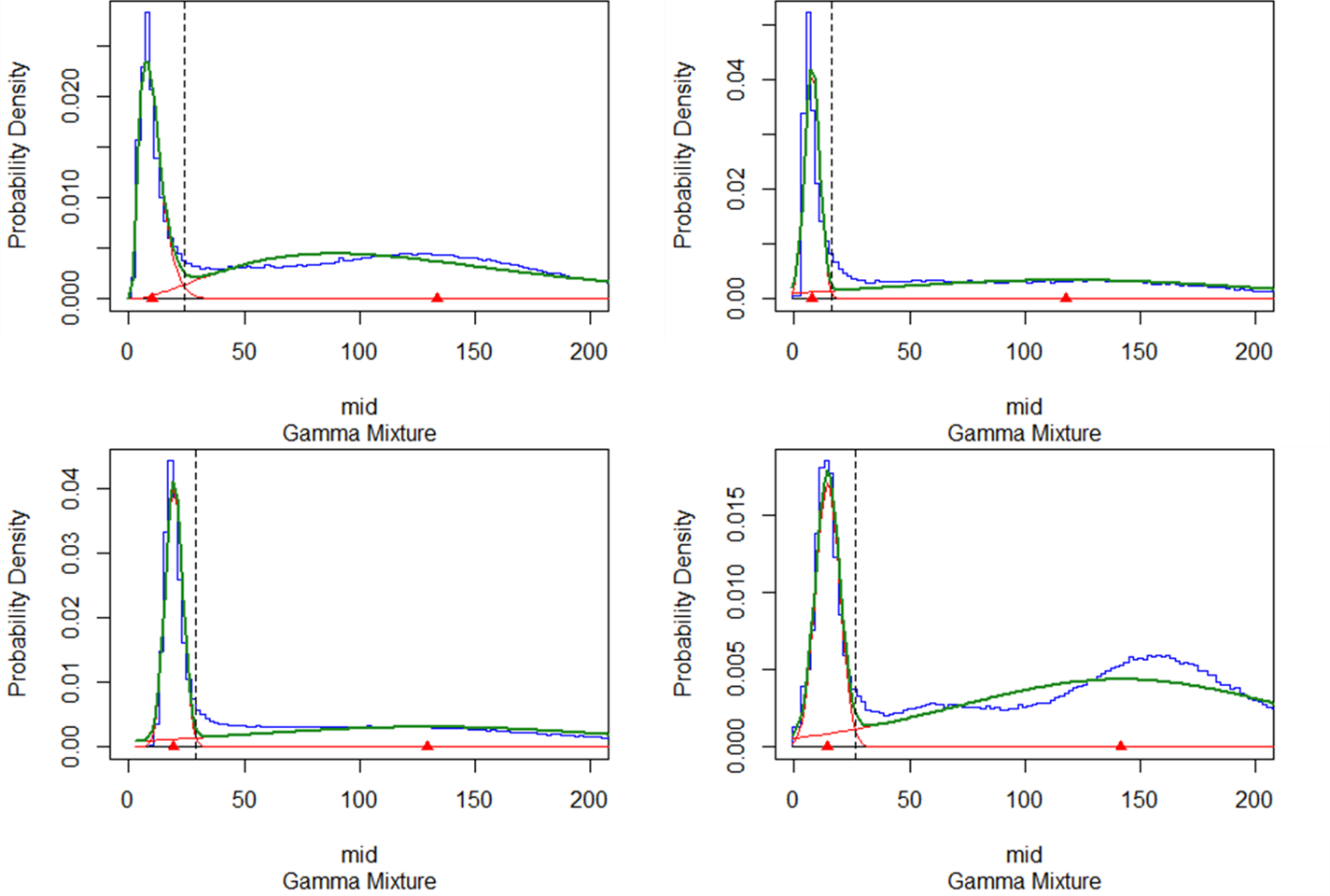


Figure S 1: Probability density histograms for the VeDBA of the four transmitter types used in this study. Blue lines show the probability density histogram (contours of bins), green lines give the corresponding probability density functions, red lines show the fitted gamma distributions. Red triangles indicate the mid points of the gamma distributions for inactive and active behaviour. Dashed vertical lines indicate the thresholds used to distinguish VeDBA values indicating active and inactive behaviour (24, 16.5, 29 and 26.5 respectively). VeDBA for flying behaviour is much higher, and its gamma distribution does not overlap with the distribution for active behaviour. For all transmitters a threshold of VeDBA > 550 was used.


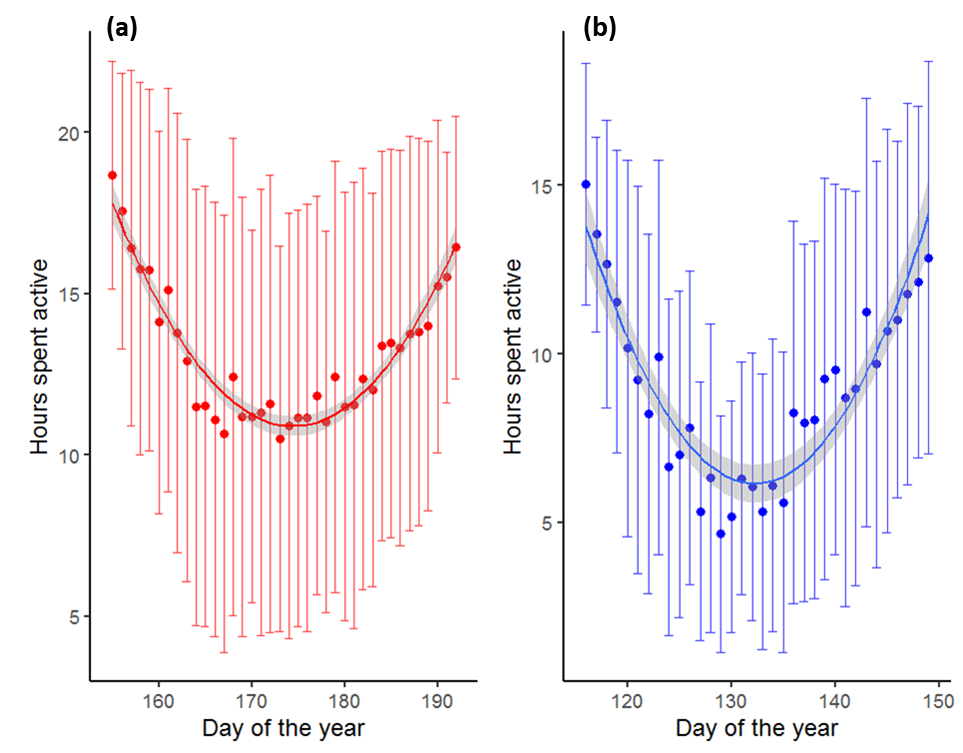


Figure S 2: Mean daily activity (mean ± SD) during the incubation trough of the migratory population (panel a) and resident population (panel b). Quadratic fits used to determine the moment of peak incubation are given by red (migratory geese) and blue (resident geese) lines. Migratory: y = 567.20 – 6.38x + 0.018x^2^; Resident: y = 506.83 – 7.57x + 0.029x^2^.


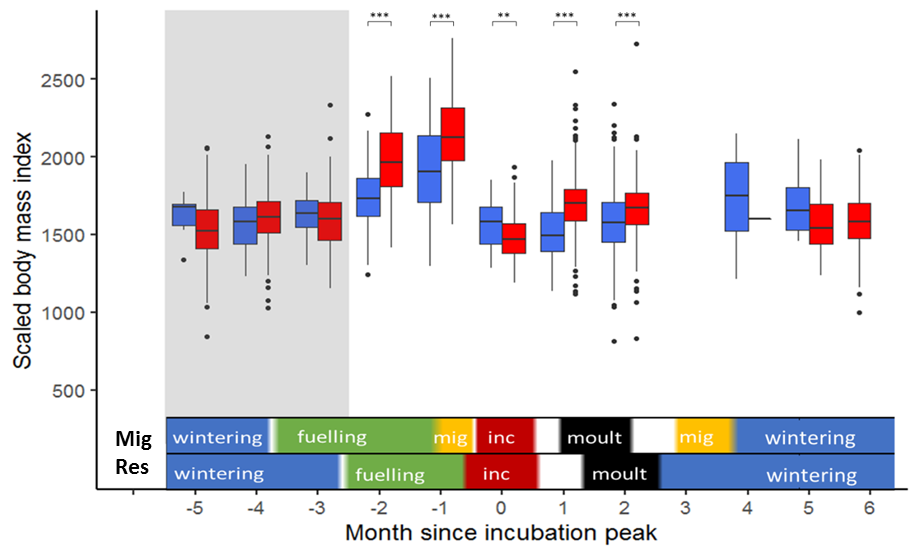


Figure S 3: Boxplot of the annual variation in body condition (scaled body mass index (Peig & Green, 2009)) for geese of the migratory (red) and resident population (blue). Significant differences between the populations are indicated per month relative to the moment of peak incubation (*: p < 0.05, **: p < 0.01, *** p < 0.001 after applying a Bonferroni correction). Boxes in the grey band indicate the period during which populations overlap in wintering distribution and patterns should be interpreted with caution. Coloured bars above the x-axis show the stage in the annual cycle of both populations (Mig: migratory, Res: resident), based on data published in van der Jeugd et al. (2009) for incubation (red) and moult (period between mean onset and end of breeding and moult ± 1 SD). Migration periods (yellow) are estimated based on the GPS data. Fuelling and wintering are estimated as the periods between stages with known timing. Blanks indicate uncertainty on life stage.

*References*

Fauna Beheer Eenheid Zuid-Holland (2021) *Faunabeheerplan ganzen Zuid-Holland 2022-2027*.

van der Jeugd, H.P., Eichhorn, G., Litvin, K.E., Stahl, J., Larsson, K., van Der Graaf, A.J. & Drent, R.H. (2009) ‘Keeping up with early springs: rapid range expansion in an avian herbivore incurs a mismatch between reproductive timing and food supply’, *Global Change Biology*, 15(5), pp. 1057–1071.

Peig, J. & Green, A.J. (2009) ‘New perspectives for estimating body condition from mass/length data: The scaled mass index as an alternative method’, *Oikos*, 118(12), pp. 1883–1891. doi: 10.1111/j.1600-0706.2009.17643.x.
